# Effect of Mobilisation With Movement on Sacroiliac Joint Dysfunction in Postnatal Women

**DOI:** 10.7759/cureus.111281

**Published:** 2026-06-22

**Authors:** Neha Pisal, Mebin S Thomas, Sandeep Shinde, Akshaya V Joshi, Sanjaykumar Patil

**Affiliations:** 1 Department of Musculoskeletal Sciences, Krishna College of Physiotherapy, Krishna Vishwa Vidyapeeth (Deemed to be University), Karad, IND; 2 Department of Obstetrics and Gynaecology, Krishna College of Physiotherapy, Krishna Vishwa Vidyapeeth (Deemed to be University), Karad, IND

**Keywords:** core stabilization, force closure, iliosacral dysfunction, innominate rotation, mobilisation, postpartum period

## Abstract

Background

Postnatal sacroiliac joint dysfunction (SIJD) frequently persists into the extended postpartum period due to altered biomechanics and residual ligamentous laxity. This study investigated the preliminary clinical efficacy of incorporating Mulligan’s Mobilisation With Movement (MWM) into a progressive conventional stabilisation program versus conventional stabilisation alone in women beyond six months postpartum.

Methods

In this comparative study, 102 postnatal women following lower segment caesarean section (LSCS) (aged 20-35 years, > 6 months postpartum) with clinically confirmed SIJD were randomly allocated to Group A (MWM plus conventional stabilisation, n = 51) or Group B (conventional stabilisation alone, n = 51) for an eight-week intervention protocol. Outcome measures included the Visual Analogue Scale (VAS), Timed Up and Go (TUG) test, and bi-directional lumbo-pelvic range of motion (flexion and extension).

Results

Baseline clinical and demographic characteristics were homogeneous between cohorts (p > 0.05). Post-intervention, Group A demonstrated more pronounced improvements than Group B across all parameters (p < 0.001). Group A achieved a mean VAS pain reduction of 4.70 points compared to 2.65 points in Group B. Functional mobility improved with a 5.40-second TUG reduction in Group A versus 2.90 seconds in Group B. Furthermore, bi-directional mobility gains were greater in Group A, with flexion expanding by 25.70° and extension by 14.10°.

Conclusion

These preliminary findings suggest that combining directional-specific MWM with conventional stabilisation exercises provides meaningful short-term clinical benefits. This integrated approach may offer a valuable mechanical strategy for reducing pain, improving dynamic stability, and restoring mobility in late postnatal SIJD.

## Introduction

The sacroiliac joint (SIJ) functions as a fundamental shock-absorbing architecture within the human frame, transferring multidirectional physiological loading between the axial column and the appendicular lower extremities [1]. Because it is a diarthrodial synovial articulation with highly irregular, reciprocal articular surfaces, its mechanical stability relies on a delicate balance of architectural form closure and dynamic force closure [2]. Form closure is provided by the structural congruency of the sacrum and the ilia, reinforced by dense posterior, anterior, and interosseous ligamentous complexes. Conversely, force closure is regulated by synchronised, dynamic myofascial systems including the transverse abdominis (TA), lumbar multifidus, gluteal complex, and thoracolumbar fascia, which generate compression across the joint planes to handle active kinetic demands [1,2].

During pregnancy and the subsequent postpartum period, this intricate stability framework faces substantial structural and neuro-mechanical disruption [2,3]. While up to 65% of women report some degree of peripartum pelvic ring discomfort, the natural history of pelvic girdle issues shows that spontaneous resolution typically occurs within the first six to 12 weeks postpartum as gestational hormone levels, particularly relaxin and progesterone, decline. However, longitudinal epidemiological data reveal that approximately 10% to 18% of women experience non-resolving, persistent symptoms that extend well into the late postpartum period [3]. In clinical practice, if mechanical tracking errors and pain fail to resolve spontaneously by the six-month postpartum milestone, the condition is classified as chronic or persistent postpartum pelvic girdle dysfunction, as the curve for spontaneous recovery plateaus after this point [4]. Choosing a selection threshold of greater than six months postpartum is therefore structurally vital to isolate established, non-resolving sacroiliac joint dysfunction (SIJD) from transient, self-limiting early postnatal alterations.

The pathomechanics of postnatal SIJD are frequently characterised by asymmetric tracking mismatches of the innominate bones relative to the sacrum, typically manifesting as persistent anterior or posterior innominate rotational faults [5]. When residual hormonal laxity prevents these bones from returning to their neutral anatomical alignment, the surrounding ligaments are subjected to prolonged, uneven tensile stress. This continuous mechanical strain triggers a chronic cascade of localised micro-trauma, neurogenic inflammation, and active nociceptive firing within the highly innervated periarticular tissues [5]. In response to this pain, the local neuromuscular system often initiates protective muscle guarding or hypertonicity in surrounding structures like the piriformis or quadratus lumborum, creating a cyclical pattern of structural locking and movement compensation [3,5].

Crucially, the pathomechanical presentation of persistent postpartum SIJD is heavily influenced by the mode of delivery. While vaginal births primarily stretch the posterior pelvic ligaments and pelvic floor musculature, a lower segment caesarean section (LSCS) introduces unique, localised soft-tissue trauma [6]. An LSCS requires a horizontal transverse abdominal incision (e.g., Pfannenstiel or Joel-Cohen techniques) that cuts or divides the skin, subcutaneous layers, and rectus sheath, while separating the fascial anchor points of the deepest core stabiliser, the transversus abdominis (TrA), and the internal oblique muscles. This direct surgical trauma can disrupt the abdominal myofascial planes, create restrictive scar tissue, and impair optimal TrA activation. Consequently, the active anterior force closure mechanism of the pelvic ring is compromised, leaving the hypermobile sacroiliac surfaces vulnerable to persistent tracking errors [7]. By restricting our cohort exclusively to women post-LSCS, we eliminated the biomechanical variability associated with vaginal deliveries (such as pelvic floor trauma or pudendal nerve injury). Our findings specifically highlight the efficacy of MWM in addressing SIJD caused by this surgical disruption of the TrA and anterior abdominal fascia, which severely compromises the physiological force closure of the SIJ.

These altered biomechanics directly impact basic functional capacity and everyday activities of daily living (ADLs) in postpartum women. Postnatal individuals with SIJD frequently exhibit noticeable deficits in weight transfer, asymmetric loading during gait, and significant functional limitations during transitional movements like sit-to-stand changes, turning, or lifting and carrying an infant [8]. The disruption of optimal force closure means that tasks requiring coordinated core stabilisation instead cause mechanical shearing forces across the unstable sacroiliac surfaces, leading to early physical fatigue, altered balance parameters, and a high risk of developing persistent, chronic pain conditions [3,8].

Despite the substantial functional burden associated with postpartum pelvic girdle issues, optimal clinical management remains a subject of ongoing discussion within physical rehabilitation. Conventional physical therapy programs historically prioritise active force closure strategies, incorporating localised muscle strengthening exercises such as gluteal bridges or core bracing combined with passive modalities or stretching to address muscular imbalances [9]. However, emerging clinical trials suggest that relying solely on strengthening without correcting the underlying articular positional fault can yield limited results, as the active stabilisation system continues to pull against a mechanically locked joint frame [8,9]. Consequently, there is a clear clinical need for integrated manual interventions that directly address both the articular alignment fault and the supporting muscular framework to achieve comprehensive functional recovery.

Mulligan’s Mobilisation With Movement (MWM) offers an alternative manual approach designed to break this chronic pathomechanical cycle. Based on the positional fault hypothesis, MWM addresses structural tracking errors by combining a sustained, passive manual accessory glide applied by the clinician with an active, physiological movement performed by the patient [10]. This concurrent application aims to eliminate joint restriction, correct localised intra-articular tracking mismatches, and restore normal neuro-mechanical output. However, it must be recognised that current high-quality evidence demonstrating the clinical efficacy of manual joint centration via MWM is derived almost entirely from general orthopaedic populations presenting with mechanical low back pain, rather than individuals with postpartum pelvic ring issues [10,11]. There remains a complete gap in the literature regarding whether these joint mobilisation strategies can successfully overcome the combined structural challenges of ligamentous laxity and post-surgical abdominal scarring seen in postpartum individuals.

To address this gap in the literature, this prospective, comparative study was designed to evaluate the clinical effects of integrating directional-specific MWM with a conventional rehabilitation protocol. The primary outcome of this investigation was pain intensity, measured objectively via the Visual Analogue Scale (VAS). The secondary outcomes included functional mobility, assessed using the Timed Up and Go (TUG) test, and lumbo-pelvic range of motion (ROM) evaluated for both forward flexion and extension planes. Hence, this study aimed to compare the clinical efficacy of incorporating Mulligan’s MWM into a progressive conventional stabilisation program versus conventional stabilisation alone on pain intensity (VAS), functional mobility (TUG), and lumbo-pelvic ROM (flexion and extension) in primiparous women presenting with SIJD beyond six months post-caesarean section (LSCS).

## Materials and methods

This investigation is designed as a prospective, comparative study evaluating the therapeutic effects of MWM compared to conventional therapy in postnatal women with SIJD. The research was carried out from 2nd September 2025 to 30th April 2026.

The sample size for this investigation was determined a priori using a power analysis based on the primary outcome measure, the VAS. The calculation was conducted to detect a clinically meaningful minimum difference between groups, securing an alpha level (α) of 0.05 and a statistical power (1-β) of 80%. The following formula was utilised: $N = \frac{(SD_{1}^{2} + SD_{2}^{2})(Z_{1-\alpha/2} + Z_{1-\beta})^{2}}{(\bar{x}_{1} - \bar{x}_{2})^{2}}$. The specific input parameters utilised included an expected mean between-group change difference $(\bar{x}_{1} - \bar{x}_{2})$ of 0.70 points, paired with variance estimators (SD1 = 1.15 and SD2 = 1.20) modelled from prior manual therapy trials for SIJD [12]. The computation indicated a base requirement of approximately 46 participants per group (92 total). To account for an anticipated 10% participant attrition rate over the intervention period, the final recruitment target was established at 102 participants, resulting in 51 participants randomly allocated to each respective treatment arm.

**Figure 1:**
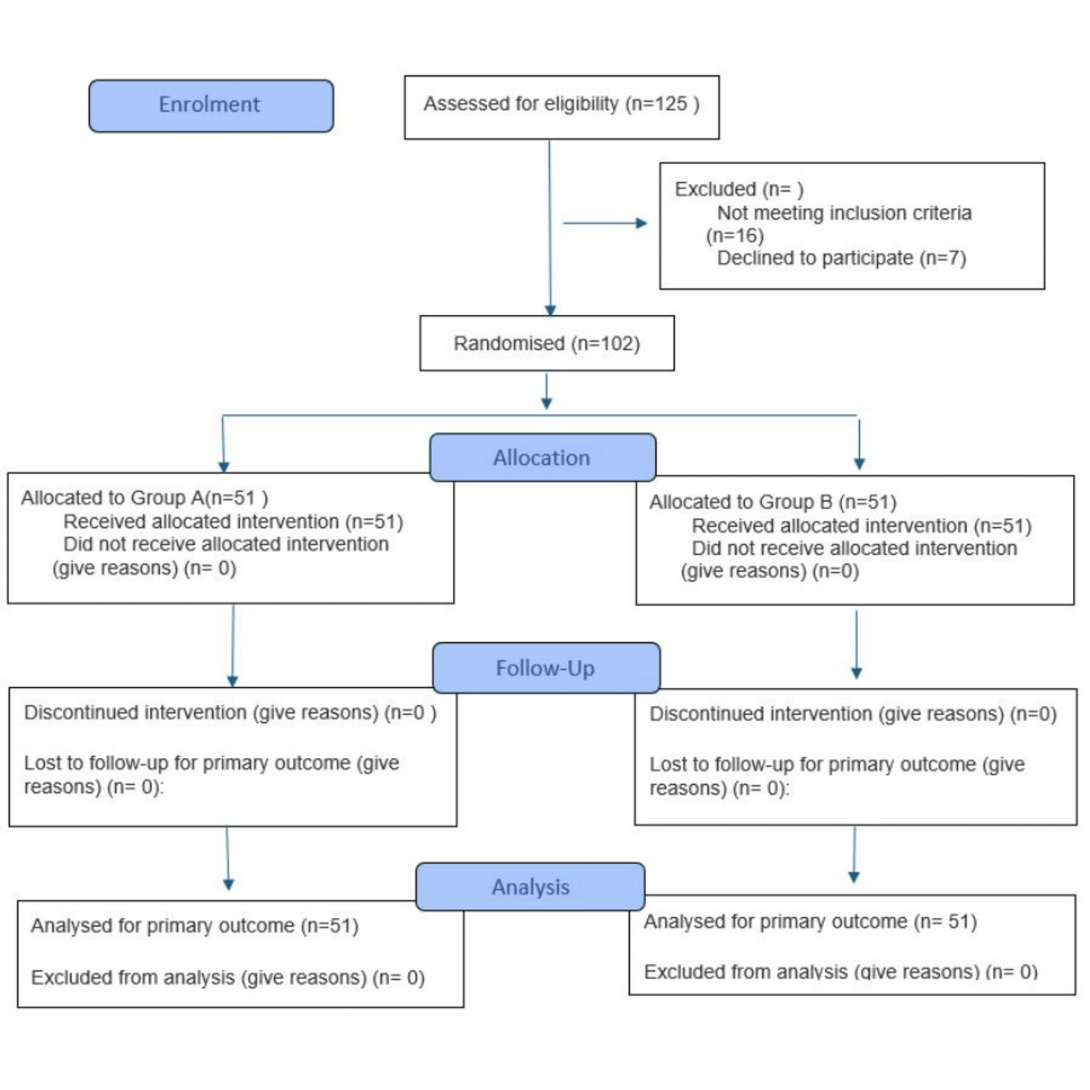
CONSORT flow diagram CONSORT: Consolidated Standards of Reporting Trials

A total of 125 postpartum women presenting with lumbo-pelvic discomfort were initially screened for eligibility in the clinical department. Following this preliminary screening funnel, 23 individuals were excluded from the trial, of which six presented with clinical signs of active lumbar radiculopathy or discogenic centralisation, four had a documented history of prior lumbar or pelvic surgery, three had received localised corticosteroid injections within the past three months, and three failed to reach the required minimum threshold of three positive signs on the five-test provocative cluster. The remaining seven individuals who declined participation cited personal scheduling constraints or transportation limitations (Figure 1).

Eligible individuals were primiparous postnatal women aged between 20 and 35 years who had undergone a LSCS and were beyond six months postpartum. Clinically, they were required to demonstrate a VAS pain score greater than 3/10 with symptom duration exceeding four weeks. Additionally, a definitive clinical diagnosis of SIJD was established by localised mechanical tenderness over the posterior superior iliac spine (PSIS) and a minimum of three positive responses out of a five-test provocative clinical cluster comprising the thigh thrust test, distraction test, compression test, Gaenslen’s test, and sacral thrust test [13].

The distraction test involves applying outward pressure on the anterior superior iliac spines (ASIS). The compression test involves applying downward pressure on the iliac crests in a side-lying position. The thigh thrust test involves applying a posterior shearing force through the femur. Gaenslen’s test involves applying torsional stress to the pelvis by flexing one hip and extending the contralateral hip. The sacral thrust test involves applying an anteriorly directed force over the sacrum in a prone position. Additionally, repeated lumbar movements and the Flexion, Abduction, and External Rotation (FABER) test will be used to rule out primary discogenic or hip joint pathologies.

The FABER test (κ = 0.78; PABAK = 0.92) was integrated strictly as a differential diagnostic screening mechanism to isolate and exclude primary hip joint pathology rather than to confirm sacroiliac involvement [14]. During execution, the participant lay supine while the examiner placed the testing limb into a figure-four position, resting the lateral malleolus on the contralateral thigh just proximal to the patella. The examiner stabilised the opposite anterior superior iliac spine while applying a gentle, downward overpressure to the medial aspect of the flexed knee. The explicit cutoff threshold for immediate exclusion was defined as the reproduction of acute structural pain localised to the anterior hip, groin, or a clear mechanical restriction in hip capsular mobility, characterised by an inability to lower the testing knee parallel to the horizontal plane of the examination table or a measured abduction angle of less than 30°. Any participant meeting these specific cutoff criteria was interpreted as presenting with co-existing hip joint pathology and was immediately excluded from the study sample to preserve data homogeneity.

Rigid exclusion criteria were applied to eliminate confounding structural or systemic conditions. Patients presenting with active lumbar radiculopathy or disc pathology were ruled out if repeated lumbar flexion and extension movements elicited centralisation or peripheralisation of symptoms. Further exclusions comprised individuals who underwent normal vaginal delivery (NVD), instrumental vaginal delivery (forceps or vacuum-assisted), or vaginal birth after caesarean (VBAC), or those who had received localised corticosteroid injections within the preceding three months. To isolate the therapeutic effects of the study interventions, strict criteria governing permitted and prohibited concomitant treatments were enforced. Participants were prohibited from using pelvic support belts, external lumbar binders, concurrent external physical therapy protocols, or independent fitness programs throughout the eight-week timeline. Pharmacological analgesics or non-steroidal anti-inflammatory drugs (NSAIDs) were strictly restricted, requiring an absolute 48-hour washout period prior to any scheduled baseline or post-intervention outcome measurement session. Additionally, baseline screening accounted for postpartum-specific physiological variables. Obstetric triage included the assessment of lactation status, pregnancy screening, structural pelvic floor integrity, and checking for diastasis recti abdominis (DRA). Exclusion criteria mandated the elimination of candidates presenting with a new confirmed pregnancy, severe pelvic floor dysfunction (defined as pelvic organ prolapse > Stage II or active clinical urinary incontinence), or severe, non-compensated DRA demonstrating an inter-recti distance of >3.0 cm via digital caliper assessment. Lactation distribution profiles were documented and verified to be balanced between cohorts.

Once SIJD was confirmed via the provocation cluster, the direction of the innominate fault was determined using the standing flexion test, static palpation of the ASIS/PSIS, and the supine-to-sit test [15,16]. Participants were classified as having either an anterior or posterior innominate rotation, ensuring that the MWM intervention in Group A was specifically tailored to the mechanical presentation of the individual participant.

For participants presenting with bilateral symptoms, a predefined algorithm guided the MWM sequence. The side demonstrating higher pain during weight-bearing or a stronger baseline provocation response was treated first. If tracking patterns were identical bilaterally, accessory glides were applied to both joints sequentially within the same session. If rotational directions differed between sides (e.g., an anterior fault on one side and a posterior fault on the other), direction-specific glides were customised and delivered sequentially to each joint to correct its distinct positional mismatch.

To maximise internal consistency and eliminate inter-rater variance, all clinical screening, diagnostic provocation clustering, and positional innominate fault classifications were executed by a single senior orthopaedic physical therapist who underwent dedicated training to standardise test mechanics. The five-test provocation cluster was performed in a fixed, non-varying sequence: distraction, compression, thigh thrust, Gaenslen’s test, and sacral thrust, always beginning on the asymptomatic side first. Because a single, designated clinical assessor performed all entry-level diagnostic procedures, inter-rater reliability metrics (kappa) were not applicable within this trial framework.

Following baseline evaluations, the 102 enrolled participants were randomly assigned to either Group A (n = 51), the experimental group receiving Mulligan’s MWM plus conventional stabilisation, or Group B (n = 51), the control group receiving the conventional stabilisation protocol alone. Randomisation assignments were prepared by an independent researcher and sealed within sequentially numbered, opaque, sealed envelopes. These envelopes were opened sequentially only after baseline measurements were fully recorded. Due to the physical nature of manual therapy glides and therapeutic exercises, a double-blind design was impossible. Consequently, a single-blinded, open-label design was implemented where the treating physical therapist and the outcome assessors were unblinded, but the participants remained completely blinded to their group allocation. To maintain measurement consistency, the assessors completed formal practice sessions to standardise twin-axis inclinometer alignment and digital stopwatch TUG triggering parameters prior to study inception. Participants were treated in separate time slots to prevent inter-group communication, and they remained unaware of the alternative therapeutic protocol or the study hypotheses.

Over the eight-week intervention period, the trial recorded zero dropouts, maintaining a 100% participant retention rate across both cohorts through to the final post-test data collection. Treatment adherence was monitored using daily clinic attendance sheets. To minimise data attrition and preserve methodological validity, a minimum attendance threshold of 90% was mandatory for inclusion in the final statistical analysis, which all 102 participants successfully fulfilled.

Ethical considerations

Prior to the recruitment and screening of the initial participant, formal ethical clearance was reviewed and approved by the Institutional Ethics Committee (IEC) of Krishna Vishwa Vidyapeeth (Deemed to be University), Karad (protocol number: 212/2025-2026; date: 2/09/2025). Conducted in adherence to the ethical standards established by the Declaration of Helsinki, this study prioritises the protection of all human subjects. Before formal entry into the trial, all potential participants were fully informed of the research scope and subsequently provided voluntary, written informed consent. The clinical demonstration photographs displayed within this manuscript depict a healthy demonstration volunteer rather than an active study participant. Prior to capturing the photographs, explicit, written informed consent for open-access publication was obtained from the volunteer, satisfying core privacy mandates despite facial pixelation.

Outcome measures

VAS

VAS was utilised as the primary subjective outcome metric to evaluate localised pain intensity on a 0-10 continuous millimetre scale, utilising the classic, primary validation framework established by Huskisson [17].

ROM

ROM was assessed using a standardised double-inclinometer method. The superior inclinometer was positioned over the spinous process of T12, and the inferior inclinometer was placed over the sacral base (S2). The true lumbar spine range was mathematically isolated by subtracting the sacral inclination from the total gross inclination, with measurements taken in triplicate to record the average value, adhering to the original technical validation protocols defined by Saur et al. [18].

TUG Test

Employed to measure functional mobility, dynamic balancing capacity, and basic gait transitions according to the original gold-standard structural parameters validated by Podsiadlo and Richardson [19]. Participants were timed dynamically as they stood up from a standard armless chair, walked a distance of 3 m, turned around, walked back, and returned completely to a seated position.

Treatment protocol

The study was conducted over an eight-week intervention period with both groups participating in supervised physical therapy sessions three times per week (24 total sessions). Each session lasted approximately 45-60 minutes. To standardise baseline conditions and mitigate superficial muscle guarding, each session for both cohorts commenced with the application of a superficial thermal agent: a moist hot pack wrapped in protective towelling was applied to the lumbosacral and sacroiliac regions for 10 minutes. This pre-treatment thermotherapy was integrated to enhance local myofascial viscoelasticity and induce muscle relaxation [20]. In Group A, this reduction in tissue resistance facilitated optimal manual joint centration during the subsequent application of sustained accessory glides. Group B (n = 51) received a standardised conventional exercise protocol alone. Group A (n = 51) served as the experimental arm and received directionally specific Mulligan’s MWM techniques integrated with the baseline conventional protocol.

Baseline Conventional Therapy (Group A and Group B)

Both groups performed a standardised exercise program aimed at restoring lumbopelvic force closure and flexibility. This protocol is based on established clinical guidelines for postnatal sacroiliac dysfunction.

Group B (Conventional Exercise)

Following the initial 10-minute hot pack application, participants performed a progressive exercise program aimed at restoring lumbo-pelvic force closure and flexibility:

Phase I (weeks 1-2):

- TA bracing: Participants were taught to perform a gentle "hollowing" contraction of the lower abdominal wall while maintaining a neutral spine in supine and quadrupod positions (one to two sets of 10 repetitions, 10-second hold).

- Targeted stretching: Focused stretching of the hamstrings, iliopsoas, and piriformis. Each stretch was held for 30-60 seconds and repeated three times.

Phase II (weeks 3-5):

- Modified clamshell exercises: Performed in side-lying with knees flexed to 45°. Participants abducted the top knee while keeping feet together to target the gluteus medius without pelvic rotation (three sets of 15 repetitions) [10].

- Gluteal bridges: Supine hip elevation with TA bracing to improve posterior chain stability and pelvic control (three sets of 10 repetitions).

- Maintenance flexibility: Continued adherence to the targeted stretching protocol established in Phase I and cat-camel mobility (two sets of 10 repetitions).

Phase III (weeks 6-8):

- Advanced dynamic stabilisation: Progression by incorporating the quadruped bird-dog exercise alongside a single-leg glute bridge. The operational mechanics for the single-leg glute bridge were standardised as follows: the participant assumed a supine starting position with the uninjured limb's knee flexed to 90° and the foot flat on the plinth, while the contralateral limb was maintained in extended alignment parallel to the opposite thigh.

- Without manual or external support, the participant performed a concentric gluteal contraction to elevate the pelvis until a straight line was achieved from the shoulders to the active knee. This position was held statically for five seconds before returning to the starting position via controlled eccentric lowering. This movement pattern was performed for three sets of 10 repetitions per side, with progression governed by the maintenance of spinal neutrality and zero lateral pelvic drop or compensatory lumbar hyperextension.

- Movement retraining: Practice of sit-to-stand transitions and lifting mechanics, emphasising a neutral pelvic alignment and controlled bracing during functional postnatal tasks (three sets of 10 repetitions per side).

Group A (Mobilisation With Movement)

Following the initial 10-minute baseline hot pack application, and in addition to performing the baseline stabilisation and core training exercises outlined in the conventional protocol, Group A received targeted Mulligan’s MWM techniques.

**Figure 2:**
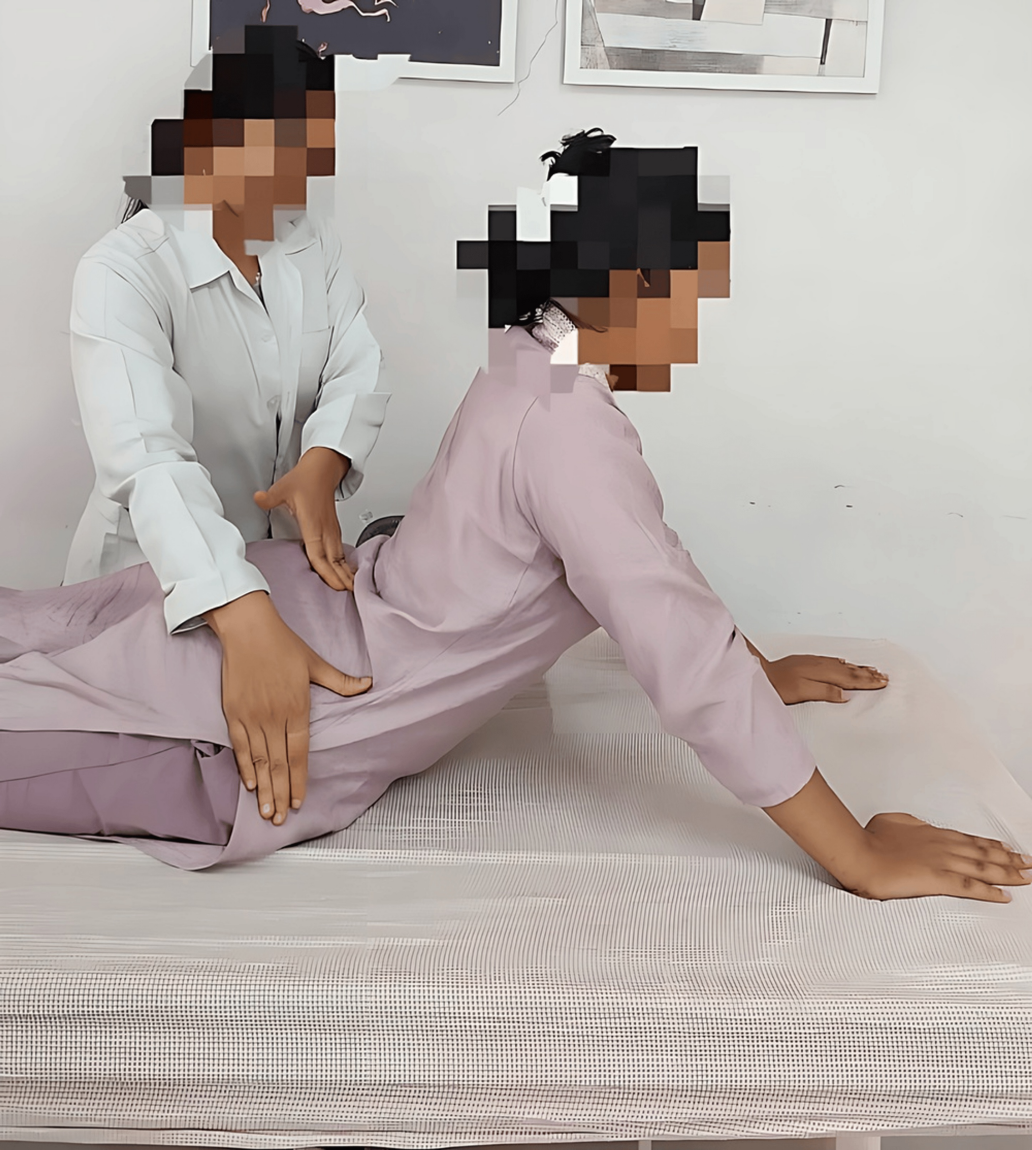
Mulligan’s Mobilisation With Movement (MWM) Clinical execution of Mulligan’s Mobilisation With Movement (MWM) for sacroiliac joint dysfunction. The treating physical therapist applies a sustained, pain-free manual accessory glide to the innominate bone while the postnatal participant concurrently performs an active physiological movement to correct the mechanical tracking error.

**Figure 3:**
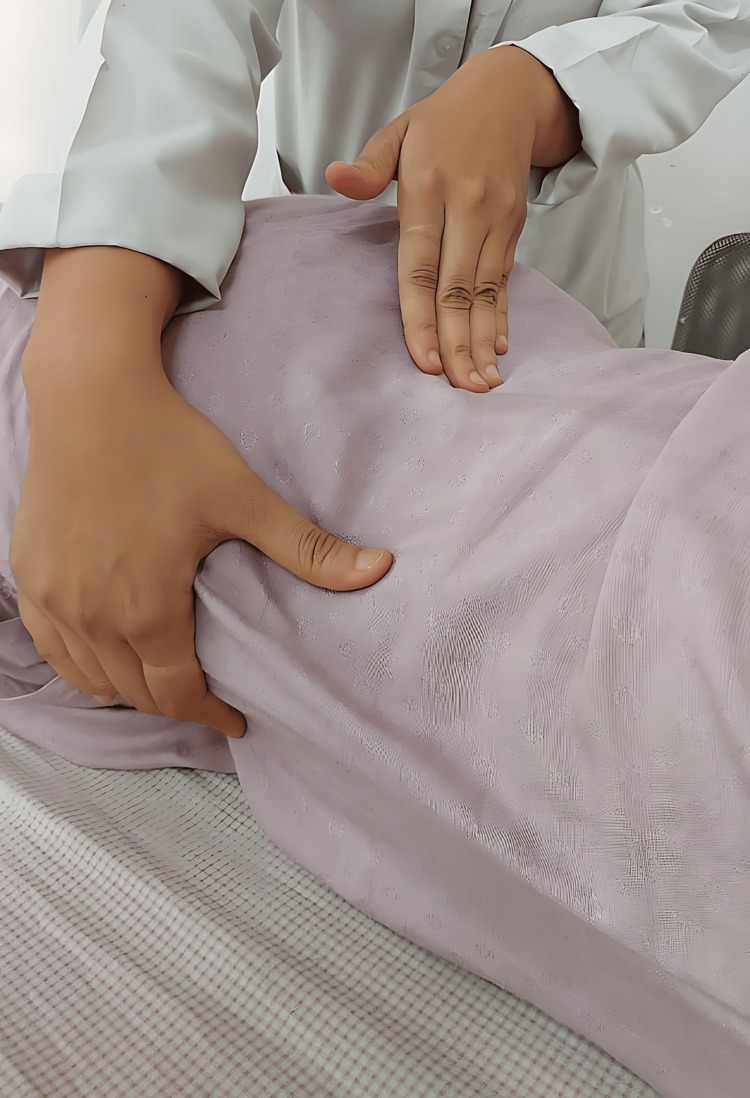
Hand placement Detailed view of the specific clinician’s hand placement and vector orientation. The therapist's stabilising hand secures the sacral base while the active mobilising hand grips the iliac crest, applying a targeted posterior-superior or anterior-inferior corrective force tailored to the participant's specific positional fault presentation.

Principles of application: The therapist applied a sustained accessory glide while the patient performed a symptomatic active movement. All glides followed the PILL (pain-free, instant result, long-lasting) principle and the CROKS (Contraindications, Repetitions, Overpressure, Knowledge, Sustained) guidelines. The overall clinical execution of the glide is illustrated in Figure 2 and Figure 3.

For Anterior Innominate Dysfunction (Posterior Glide MWM)

The patient was positioned in a side-lying or supine position. The therapist stabilised the sacrum and applied a posterior-superior glide to the ilium. While the glide was sustained, the patient actively flexed the hip (knee-to-chest) or performed a posterior pelvic tilt.

For Posterior Innominate Dysfunction (Anterior Glide MWM)

The therapist applied an anterior-inferior glide to the iliac crest. While the glide was sustained, the patient performed active hip extension or an anterior pelvic tilt.

Phase I (weeks 1-2):

- Sustained accessory glides: Depending on the specific diagnosis (anterior or posterior innominate rotation), the therapist applied a sustained posterior-superior or anterior-inferior glide to the ilium.

- Pain-free active movement: While the glide was maintained, the patient performed an active physiological movement (e.g., knee-to-chest or pelvic tilt). Following the PILL principle, the glide was adjusted until the movement was completely pain-free (one to two sets of 10 repetitions).

Phase II (weeks 3-5):

- End-range overpressure: Manual overpressure was applied at the terminal range of the glide-assisted movement to facilitate long-term neuro-physiological retention of the corrected joint alignment (three sets of 10 repetitions).

- Integrated stabilisation: MWM techniques were applied while the participant engaged in Phase II stabilisation exercises (e.g., MWM-assisted gluteal bridges) to ensure joint centration during active muscle recruitment (three sets of 10 repetitions).

Phase III (weeks 6-8):

- Self-MWM training: Participants were instructed in self-mobilisation techniques using a strap or manual pressure to replicate the therapist's corrective glide during home activities (three sets of 10 repetitions).

- Dynamic functional loading: Therapist-applied glides were integrated into higher-demand functional tasks, such as stepping or lunging, ensuring biomechanical symmetry during the loading phases of gait and daily movement (three sets of 10 repetitions).

To ensure strict differentiation between the intervention arms, home program parameters were tailored specifically to each group. Participants allocated to Group A (MWM plus conventional stabilisation) were instructed in a specialised self-MWM home technique utilising manual pressure or an unyielding stabilisation strap to replicate and reinforce the direction-specific therapeutic glides performed in the clinic. Conversely, participants assigned to Group B (conventional stabilisation alone) received home training instructions restricted to basic spinal flexibility exercises and core stabilisation manoeuvres (e.g., abdominal bracing, cat-and-camel drills); no self-MWM, self-correction straps, or accessory joint glide procedures were taught or permitted in Group B, preserving a clean comparative baseline. Compliance was monitored across both cohorts using a daily exercise diary to verify adherence thresholds.

Exercises were advanced from one phase to the next based on a combined paradigm of pain tolerance, once the participant could perform the baseline sets without an increase in VAS scores of >2 points and objective functional competency. Progression from Phase I to Phase II required the participant to demonstrate optimal motor control by successfully executing 10 consecutive repetitions of TA abdominal hollowing with a 10-second hold in both supine and quadruped positions without losing a neutral spine. Advancement from Phase II to Phase III required the clean execution of three sets of 15 repetitions of clamshells and gluteal bridges with symmetrical pelvic alignment and zero lumbar substitution. Exercises were progressed by increasing the number of repetitions or the difficulty of the posture (e.g., moving from supine to standing core bracing). Final outcome measures (VAS, ROM, and TUG) were formally recorded at the conclusion of the eight-week protocol.

Statistical analysis

Statistical processing was performed utilising SPSS Statistics for Windows, Version 26.0 (IBM Corp., Armonk, NY). Prior to parametric testing, the distributional assumptions of normality and homogeneity of variances were formally verified utilising the Shapiro-Wilk test and Levene’s test, respectively, confirming that all continuous metrics satisfied requirements for parametric evaluation (p > 0.05). Descriptive metrics, including means, standard deviations (SDs), frequencies, and percentages, were calculated to summarise baseline clinical and demographic features. Baseline categorical distributions between cohorts (including age brackets, BMI classifications, symptom chronicity, affected side, and innominate fault orientation) were analysed via Pearson’s chi-square (χ²) test, where minimum expected cell frequencies consistently exceeded five.

Intra-group clinical progression from baseline to the eight-week post-intervention endpoint was analysed using paired t-tests, with variability reported as the SD of the difference score (SD_diff_). Inter-group post-intervention comparisons were executed using independent samples t-tests. Standardised effect sizes for between-group comparisons were calculated using Cohen’s d and interpreted as small (0.2), medium (0.5), or large (0.8). Statistical significance was set a priori at p < 0.05. Since multiple outcome measures were recorded, it is noted that even if a conservative Bonferroni multiple-comparison correction were applied (α = 0.0125), all major findings would remain fully significant. Age, BMI, and symptom chronicity were explicitly captured as pre-defined categorical strata during clinical triage to optimise institutional documentation workflows and were maintained as categorical blocks during analysis.

## Results

The 100% participant retention rate across the eight-week trial (n = 51 per group) was driven by flexible scheduling that accommodated infant-care demands, geographical proximity to the university facility, and immediate mechanical relief acting as a compliance incentive. No serious adverse events or dropouts occurred. Minor, self-limiting reactions included mild, localised sacroiliac soreness for four participants in Group A (7.8%) after manual overpressure, which resolved within 12 hours. Additionally, transient muscle fatigue in the gluteal and abdominal walls was reported by nine participants in Group A and 11 in Group B during the initial two weeks; this resolved within 24 hours as muscle conditioning improved.

The baseline demographic and clinical characteristics included age, body mass index (BMI), symptom duration, affected side, and innominate rotation, which were recorded for all 102 participants. Most participants were aged between 26 and 32 years (41.2% in Group A and 39.2% in Group B), representing the most active childbearing demographic. A significant majority were classified as overweight (BMI >25.0 kg/m²), accounting for 66.7% of Group A and 62.7% of Group B. Statistical analysis utilising the chi-square test revealed no significant between-group differences for age (p = 0.97), BMI (p = 0.67), or side of symptoms (p = 0.9). Furthermore, symptom chronicity was highly comparable, with 52.9% of Group A and 51.0% of Group B reporting symptoms lasting between four and 12 weeks (p = 0.84). The clinical presentation of innominate rotation was also well-balanced (p = 0.85), with nearly equal distribution between anterior and posterior rotational faults in both groups, ensuring that both groups started from a statistically similar baseline. Group A presented a mean time since delivery of 9.41 ± 2.14 months (minimum: 6.0 months; maximum: 17.0 months), while Group B demonstrated a mean time since delivery of 9.58 ± 2.31 months (minimum: 6.0 months; maximum: 18.0 months). Independent samples t-test evaluation confirmed that this parameter was statistically homogeneous between groups (t = -0.385, p = 0.701), indicating no baseline imbalance in the natural recovery timeline context. The comprehensive baseline demographic profiles and clinical characteristics of the study cohorts are detailed in Table 1.

**Table 1:** Demographic variables of participants Categorical variables are expressed as absolute frequencies accompanied by relative percentages in parentheses: n (%). Continuous variables are expressed as mean ± standard deviation (SD). Baseline inferential homogeneity mapping was performed using two distinct comparative methodologies based on data-structure boundaries; all cell counts met the assumption of expected frequencies > 5. A p-value of >0.05 indicates no statistically significant difference between the groups at baseline. *Reported as median (interquartile range (IQR)) due to non-normal distribution; evaluated via Mann-Whitney U test (Z). Group A: MWM (n = 51); Group B: conventional exercise (n = 51). MWM: Mobilisation With Movement

Variable	Group A (n = 51)	Group B (n = 51)	Chi-square (χ²) value	p-value
Age (years, categories)			0.053	0.97
20-25	17 (33.3%)	18 (35.3%)	-	-
26-32	21 (41.2%)	20 (39.2%)	-	-
33-35	13 (25.5%)	13 (25.5%)	-	-
Age (continuous, mean ± SD)	27.8 ± 3.9	27.6 ± 4.1	0.251	0.80
Body mass index (BMI) (categories)			0.172	0.67
Normal weight (< 25.0)	17 (33.3%)	19 (37.3%)	-	-
Overweight (>25)	34 (66.7%)	32 (62.7%)	-	-
BMI (continuous, mean ± SD)	24.1 ± 2.2	23.9 ± 2.4	0.438	0.66
Duration of chronicity (categories)			0.039	0.84
4-12 weeks	27 (52.9%)	26 (51.0%)	-	-
> 12 weeks	24 (47.1%)	25 (49.0%)	-	-
Duration of chronicity (weeks)*	14.5 (9.0, 20.0)	15.0 (10.0, 22.0)	Z = 0.31	0.75
Time since delivery (months) (mean ± SD) (range)	9.41 ± 2.14 (6.0-17.0)	9.58 ± 2.31 months (6.0-18.0)	t = -0.385	0.70
Side of symptoms			0.049	0.97
Unilateral (right)	22 (43.1%)	21 (41.2%)	-	-
Unilateral (left)	19 (37.3%)	20 (39.2%)	-	-
Bilateral	10 (19.6%)	10 (19.6%)	-	-
Innominate rotation			0.039	0.67
Anterior innominate	26 (51.0%)	25 (49.0%)	-	-
Posterior innominate	25 (49.0%)	26 (51.0%)	-	-

The statistical evaluation utilised paired t-tests to determine the absolute effectiveness of each specific intervention over the eight-week period. The within-group analysis comparing pre-test and post-test scores for both groups is detailed in Table 2.

**Table 2:** Within-group comparison of clinical outcomes Data are presented as mean ± standard deviation (SD). Mean change values represent the pre-to-post intervention difference. The associated (±) represents the true standard deviation of the difference scores (SD_diff_), and 95% CI represents the confidence interval of the mean difference; both parameters serve as the exact mathematical basis for the within-group paired t-test calculations Group A: MWM (n = 51); Group B: conventional exercise (n = 51) MWM: Mobilisation With Movement; ROM: Range of Motion; TUG: Timed Up and Go; VAS: Visual Analogue Scale

Outcome measure	Group	Pre-test mean ± SD	Post-test mean ± SD	Mean improvement (± SD_diff_)	95% CI	t-value	p-value
VAS	Group A (MWM)	7.20 ± 1.15	2.50 ± 0.85	4.70 ± 2.31	(4.05, 5.35)	14.52	<0.001
	Group B (Conventional)	7.15 ± 1.20	4.50 ± 1.10	2.65 ± 2.30	(2.00, 3.30)	8.24	<0.001
TUG (seconds)	Group A (MWM)	14.65 ± 2.45	9.25 ± 1.55	5.40 ± 3.40	(4.44, 6.36)	11.35	<0.001
	Group B (Conventional)	14.45 ± 2.50	11.55 ± 1.95	2.90 ± 3.05	(2.04, 3.76)	6.78	<0.001
ROM: flexion (°)	Group A (MWM)	42.50 ± 5.40	68.20 ± 4.85	25.70 ± 14.27	(21.69, 29.71)	12.86	<0.001
	Group B (Conventional)	43.10 ± 5.25	54.60 ± 5.50	11.50 ± 11.07	(8.39, 14.61)	7.42	<0.001
ROM: extension (°)	Group A (MWM)	12.40 ± 3.10	26.50 ± 2.80	14.10 ± 9.20	(11.51, 16.69)	10.95	<0.001
	Group B (Conventional)	12.80 ± 3.30	18.20 ± 3.00	5.40 ± 6.56	(3.56, 7.24)	5.88	<0.001

In Group A (MWM), the protocol was associated with significant modifications across all evaluated parameters (p < 0.001). The mean pain intensity (VAS) demonstrated a reduction of 4.70 points, while functional mobility scores showed a 5.40-second mean reduction in TUG times. The application of directional-specific MWM glides in Group A was accompanied by notable changes in both flexion (mean increase of 25.70°) and extension (mean increase of 14.10°).

In Group B (conventional therapy), the within-group analysis also demonstrated statistically significant changes (p < 0.001), with a mean reduction of 2.65 points in VAS scores, a 2.90-second improvement in TUG times, a mean increase of 11.50° in flexion, and a 5.40° increase in extension. To evaluate the homogeneity of these variance scores between the two groups, Levene's test was executed on the mean improvements; for the primary VAS outcome, Levene's test confirmed equal variances across groups (F = 0.48, p = 0.49), justifying the utilisation of standard parametric independent t-tests for subsequent between-group analysis.

At baseline, independent t-tests confirmed there were no statistically significant differences between the two groups across any outcome measure (p > 0.05), ensuring a comparable starting point for evaluating the interventions. The between-group analysis comparing the therapeutic effectiveness of Group A (MWM) and Group B (conventional therapy) across all variables is consolidated in Table 3.

**Table 3:** Between-group comparison of clinical outcomes Data are presented as mean ± standard deviation (SD). For “mean improvement” rows, values represent the pre-to-post change ± the true standard deviation of the difference scores (SD_diff_). “Mean difference (A-B)” represents the between-group difference alongside its 95% confidence interval. All p-values and t-values in this table are derived from Independent Samples t-tests. Cohen’s d represents the pooled effect size. Group A: MWM (n = 51); Group B: conventional exercise (n = 51) MWM: Mobilisation With Movement; ROM: Range of Motion; TUG: Timed Up and Go; VAS: Visual Analogue Scale

Outcome measure and parameter	Group A (MWM)	Group B (conventional)	Mean difference (A-B) (95% CI)	t-value	p-value	Cohen’s d
Visual Analogue Scale (VAS)						
Pre-test mean ± SD	7.20 ± 1.15	7.15 ± 1.20	0.05 (-0.41, 0.51)	0.21	0.831	0.04
Post-test mean ± SD	2.50 ± 0.85	4.50 ± 1.10	-2.00 (-2.38, -1.62)	10.26	<0.001	2.03
Mean improvement (pre-post)	4.70 ± 2.31	2.65 ± 2.30	2.05 (1.14, 2.96)	4.49	<0.001	0.89
Timed Up and Go (TUG) (sec)						
Pre-test mean ± SD	14.65 ± 2.45	14.45 ± 2.50	0.20 (-0.76, 1.16)	0.41	0.682	0.08
Post-test mean ± SD	9.25 ± 1.55	11.55 ± 1.95	-2.30 (-2.99, -1.61)	6.61	<0.001	1.31
Mean improvement (pre-post)	5.40 ± 3.40	2.90 ± 3.05	2.50 (1.23, 3.77)	3.91	<0.001	0.77
ROM: flexion (°)						
Pre-test mean ± SD	42.50 ± 5.40	43.10 ± 5.25	-0.60 (-2.68, 1.48)	0.57	0.570	0.11
Post-test mean ± SD	68.20 ± 4.85	54.60 ± 5.50	13.60 (11.58, 15.62)	13.33	<0.001	2.62
Mean improvement (pre-post)	25.70 ± 14.28	11.50 ± 11.07	14.20 (9.18, 19.22)	5.61	<0.001	1.11
ROM: extension (°)						
Pre-test mean ± SD	12.40 ± 3.10	12.80 ± 3.30	-0.40 (-1.64, 0.84)	0.63	0.530	0.12
Post-test mean ± SD	26.50 ± 2.80	18.20 ± 3.00	8.30 (7.17, 9.43)	14.56	<0.001	2.86
Mean improvement (pre-post)	14.10 ± 9.19	5.40 ± 6.56	8.70 (5.57, 11.83)	5.51	<0.001	1.09

Following the eight-week protocol, post-intervention analysis indicated that Group A demonstrated greater changes compared to Group B. For pain intensity (VAS), the experimental group exhibited a mean reduction that was 2.05 points greater than the control group (p < 0.001). Similarly, notable between-group differences were observed in functional outcomes, where Group A demonstrated a greater mean change of 2.50 seconds in the TUG test compared to Group B (p < 0.001).

The recovery of lumbo-pelvic ROM also appeared more extensive in the MWM cohort, with Group A demonstrating a greater mean change of 14.20° in lumbo-pelvic flexion (p < 0.001) and 8.70° in lumbo-pelvic extension (p < 0.001) relative to Group B. These preliminary findings suggest that combining directional-specific MWM with conventional stretching and stabilisation may offer a more effective intervention trend for postnatal women with SIJD than conventional therapy alone.

## Discussion

The primary objective of the current investigation was to evaluate the clinical effects of Mulligan’s MWM compared to conventional physiotherapy in postnatal women presenting with SIJD. The results indicate that while both interventions provided statistically significant improvements, the integration of directional-specific MWM (Group A) was associated with greater improvements in pain modulation, functional gait, and bi-directional lumbo-pelvic mobility. These findings suggest that mechanical realignment may be a meaningful component of functional recovery in the postnatal population.

A notable observation in this study was the more pronounced reduction in pain intensity (VAS) within the MWM Group A (p < 0.001), which exhibited a mean improvement of 2.05 points more than the control group. The pathomechanical basis for this finding likely involves the immediate correction of minor positional faults at the articular surface. During the gestational and early postnatal periods, the maternal endocrine system releases substantial quantities of relaxin and progesterone. These hormones systematically induce ligamentous laxity to facilitate the structural expansion of the birth canal for parturition. The persistence of pregnancy-related pelvic girdle pain into the postpartum period remains a significant clinical challenge. According to the Clinical Practice Guidelines established by Clinton et al., mechanical imbalances, increased ligamentous laxity, and altered motor control strategies initiated during the antepartum phase serve as strong prognostic factors for ongoing postnatal disability [21].

While the conventional exercise group of this study aimed to restore optimal lumbopelvic force closure as advocated by current pelvic health guidelines [21], our findings indicate that integrating positional-fault corrections via Mulligan's MWM glides yields a more pronounced acceleration in functional execution. The outcomes observed in Group A are consistent with the broader orthopaedic findings of Varghese et al. [22], which suggested that MWM provides an immediate mechanical "reset" by utilising a sustained, pain-free accessory glide during active, physiological movement. By restoring optimal arthrokinematics, the intervention allows for immediate pain-free movement replication, which effectively helps break the cycle of protective muscle guarding and localised tissue ischemia. Furthermore, Yan et al. [23] demonstrated that integrating manual mobilisation with core stabilisation strategies optimises outcomes for general mechanical SIJD populations. Because literature evaluating MWM specifically within persistent post-caesarean SIJD remains virtually non-existent, these general orthopaedic trials must serve as our foundational mechanistic baseline. Our study successfully fills this gap, demonstrating that these joint centration trends translate effectively to a specialised population dealing with postpartum tissue changes and surgical scarring.

To evaluate the clinical relevance of the present study outcomes, these observed changes were benchmarked against established clinical responsiveness thresholds. The minimally clinically important difference (MCID) for the VAS in postpartum pelvic girdle and mechanical low back pain environments is recognised as 2.0 points [24]. While a standalone MCID index specifically isolated for postnatal SIJD cohorts has not been separately established in the literature, translating the standard 2.0-point threshold from general mechanical low back and pelvic girdle data demonstrates that the conventional program alone (Group B) achieved a clinically meaningful benefit (2.65 points), whereas the MWM-integrated protocol (Group A) far surpassed this baseline mark with a reduction of 4.70 points.

While the clinical benefits of MWM are hypothesised to arise from the mechanical realignment of minor joint tracking errors, neurophysiological downregulation of periarticular nociceptors, and the disruption of protective muscle guarding, these pathways remain theoretical within this trial. Because this investigation did not feature immediate post-session testing, structural imaging, or electrophysiological assessments (such as electromyography or H-reflex studies), these mechanisms serve as inferred models rather than directly observed outcomes of our eight-week intervention program.

The restoration of functional mobility, evidenced by the 5.40 seconds mean improvement in the TUG test for Group A (p < 0.001), highlights a relationship between structural joint tolerance and dynamic stability. The TUG test requires a transition from a sit-to-stand task to ambulation and rapid turning, all of which place heavy functional demands across the pelvic ring. Feeney et al. [25] identified that individuals with SIJD exhibit asymmetrical gait patterns and depressed neuromuscular synergy during walking tasks. While the present study did not measure direct gait parameters, force platform metrics, or inertial sensor data, the global functional improvements captured via our TUG timing scores suggest that resolving underlying joint restrictions helps restore basic transitional mobility and dynamic loading capacity.

While Group B showed meaningful improvements through conventional stabilisation, the overall recovery in functional execution was less pronounced than in Group A. This variation suggests that combining these therapies may maximise therapeutic potential, though because this trial did not utilise a factorial design, we cannot definitively prove whether resolving mechanical restrictions must chronologically precede exercise or if the benefit was primarily driven by the higher cumulative therapeutic dose in Group A. Hypothetically, when a positional fault persists, protective muscle inhibition can occur; applying an accessory glide during movement may remove this block, facilitating more normal muscle recruitment patterns during subsequent stabilisation drills, such as the modified clamshells and gluteal bridges advocated by Jeong et al. [26]. This trend is corroborated by Rehan et al. [27] and Sivakumar et al. [28], who found that combining manual mobilisation with functional hip and core muscle activation exercises is more effective for managing pelvic girdle restrictions and TrA recruitment than isolated exercise protocols. Concurrently, the functional gains identified via the TUG test demonstrated high clinical significance. The minimal detectable change (MDC) for the TUG test within postpartum and musculoskeletal contexts ranges from 1.1 to 2.1 seconds [29]. The functional improvements recorded in both Group A (5.40 seconds) and Group B (2.90 seconds) comfortably exceeded this MDC threshold.

The bi-directional assessment of lumbo-pelvic ROM recorded notable gains in both flexion (25.70°) and extension (14.10°) within the MWM group (p < 0.001). This directional recovery is clinically relevant because postnatal SIJD rarely presents as a uniform, symmetrical restriction. Instead, it typically manifests as a specific anterior or posterior innominate rotation. An anterior positional fault typically places the posterior ligaments under prolonged tension and limits lumbo-pelvic extension, whereas a posterior positional fault restricts forward flexion. Postpartum clinical trials emphasise that generalised, non-specific stretching protocols may fail to address these distinct rotational mismatches, and in some cases of hypermobility, may even alter active pelvic floor responses and worsen joint instability [30].

The present study suggests that matching the MWM glide specifically to the diagnosed positional fault (e.g., applying a posterior glide for an anterior innominate dysfunction) helps restore global sagittal plane mobility more effectively. This directional-specific approach is supported by the biomechanical findings of Yan et al. [23], which utilised localised mobilisation techniques to target specific rotational faults and noted secondary restorations in global spinal movement. By combining MWM to address the form closure mismatch with progressive core stabilisation to address the force closure deficit, the experimental protocol aimed to achieve a more comprehensive restoration of the pelvic neutral zone.

These findings indicate that while conventional core stabilisation restores muscular force closure over time, integrating directional-specific MWM following baseline thermotherapy is vital to immediately resolve underlying arthrokinematic tracking errors. This protocol offers a targeted, mechanically sound rehabilitation pathway for primiparous post-LSCS women by mitigating superficial muscle guarding and restoring joint centration. Ultimately, this dual-layer approach provides clinicians with a predictable framework for achieving a faster, more comprehensive recovery of essential functional activities in the postpartum population.

While the data reveal statistically significant differences between the two groups, these findings should be viewed as preliminary rather than definitive. Several limitations must be considered when interpreting these outcomes. First, the study evaluated clinical outcomes immediately following the completion of an eight-week protocol; the long-term sustainability of these mechanical corrections remains to be established through future longitudinal research with six-month or 12-month follow-up intervals. Second, due to the nature of manual therapy, it was not possible to blind the treating physiotherapist, which introduces a potential for operator bias during the treatment sessions. Third, the classification of innominate faults was based entirely on clinical provocative and palpation tests rather than radiographic confirmation, which limits the ability to draw precise biomechanical conclusions regarding the exact degree of intra-articular change. Age, BMI, and symptom chronicity indices were compiled as categorical strata during preliminary intake triage to maintain clinical efficiency; thus, continuous-scale modelling was not performed. Additionally, because resource limitations required the outcome measurements to be recorded by an unblinded assessor, a potential risk of assessment bias must be acknowledged, characterising the trial as an open-label framework with blinded patients rather than a traditional single-blind structure, though this was mitigated by utilising objective digital stopwatch and mechanical inclinometer instrumentation rather than operator-dependent manual testing. This study was explicitly powered a priori to identify macro-level therapeutic discrepancies between the primary intervention arms (Group A vs. Group B). Consequently, exploratory subgroup analyses (e.g., separating outcomes by anterior vs. posterior innominate rotational directions, or tracking variations across age or BMI sub-bands) were omitted to preserve statistical power and prevent Type I errors from multiple underpowered assessments.

Furthermore, while notable bi-directional ROM gains were captured, direct linear correlation or regression analyses linking kinematic changes to subjective pain thresholds were outside the study's scope. Finally, because our sample was exclusively limited to primiparous women who underwent a LSCS, these findings specifically reflect post-surgical abdominal wall disruption. No data were collected on individuals following vaginal deliveries, meaning these clinical trends are not generalisable to non-surgical postpartum populations. Furthermore, without long-term follow-up intervals beyond eight weeks, the durability of these short-term improvements remains unknown, and we cannot determine if mechanical relapse or symptom recurrence occurs over time.

## Conclusions

This study provides preliminary evidence suggesting that the integration of Mulligan’s MWM into a conventional core stabilisation program offers potential short-term therapeutic advantages for post-caesarean women presenting with persistent SIJD. By focusing on mechanical alignment alongside muscular stabilisation, this combined strategy was associated with favourable short-term changes in pain modulation and global transitional mobility compared to conservative protocols performed in isolation within this specific surgical sample.

These initial outcomes suggest a potentially valuable clinical framework for addressing joint mechanics concurrently with progressive stabilisation exercises in post-LSCS individuals. However, because these preliminary short-term benefits were observed within a single-centre, open-label design, further large-scale, multi-centre randomised controlled trials with long-term follow-up windows remain essential to confirm these observations and establish definitive clinical guidelines for postpartum pelvic girdle care.
